# Dairying and the evolution and consequences of lactase persistence in humans

**DOI:** 10.1093/af/vfad022

**Published:** 2023-06-14

**Authors:** Jay T Stock, Jonathan C K Wells

**Affiliations:** Department of Anthropology, Social Science Centre, Western University London, Ontario, Canada; Population, Policy and Practice Research and Teaching Department, UCL Great Ormond Street Institute of Child Health, London, UK

**Keywords:** agriculture, dairying, growth, human evolution, lactase persistence

**Implications**

- The domestication of milk-producing mammals and the use of milk products has been widespread among human populations throughout the past 10,000 years.
- There has been a strong selection of genetic variants in multiple populations of Eurasia and Africa associated with lactase persistence, examples of gene-culture coevolution.
- Milk consumption provides increased calories and protein, and appears to be associated with enhanced human growth in the past and present.
- The pattern of past human evolution may constrain current cultural diversity in milk production.

## Agriculture and the Origins of Dairying

A stable and consistent food source is one of the most important evolutionary challenges facing any species. Mammalian young are typically born incapable of provisioning for themselves, but all mammalian species, through the production of milk, have evolved a unique mechanism for transferring fat, proteins, sugar, as well as immunoglobins, hormones, and nutrients to their young, fueling neural development and growth throughout infancy. Milk is an essential resource for all growing mammals until they are physically able to forage and support themselves nutritionally. It has evolved as a means for mothers to synthesize food for their young, and to provide the intestinal bacteria to optimize an infant’s absorption of nutritional resources (Hinde and German, 2012). For the majority of human societies through time and space, the nutritional role of milk, like all mammals, is related to direct maternal–infant provisioning. While human hunter-gatherers typically do not consume non-human milk or dairy products, with the domestication of ruminant animals many human societies harnessed nonhuman sources of milk for the production of secondary dairy products such as cheese and yogurt, or for direct consumption (Roffet-Salque et al., 2018). While the transition to agriculture was variable in expression based on differences in local ecology and cultural history, the origin of farming and the domestication of plants and animals had a dramatic impact on the temporal and geographic patterning of milk production and consumption across the world (Stock et al., 2023).

The transition from hunting and gathering to agricultural subsistence, often referred to as the “Neolithic Revolution,” is characterized by the development of human control over the reproduction of plants and animals, as well as their evolution through artificial selection. This involved a reduction in dietary breadth toward dependence on one or a few highly productive domesticated plants or animals, and has generally been associated with a greater proportion of dietary carbohydrates relative to protein and an increased prevalence of nutritional deficiencies (Cordain et al., 2000). The agricultural transition marks a significant change in human interaction with the natural world, where humans shifted from being predominantly influenced by natural environments to being the agents of environmental change in natural systems (Stephens et al., 2019). In this context, it is often viewed as the beginning of a series of changes in human social organization that directly result from the rise of food production and the storage of surpluses. These include sedentism, property ownership, social hierarchy, specialist craft production and related technological change, greater population density, and increased frequencies of infectious diseases and zoonotic diseases associated with domestic animals. Collectively, these changes have generally been seen to be associated with bioarcheological evidence for declining human stature and skeletal indicators of health (Larsen, 2023).

**Figure 1.**
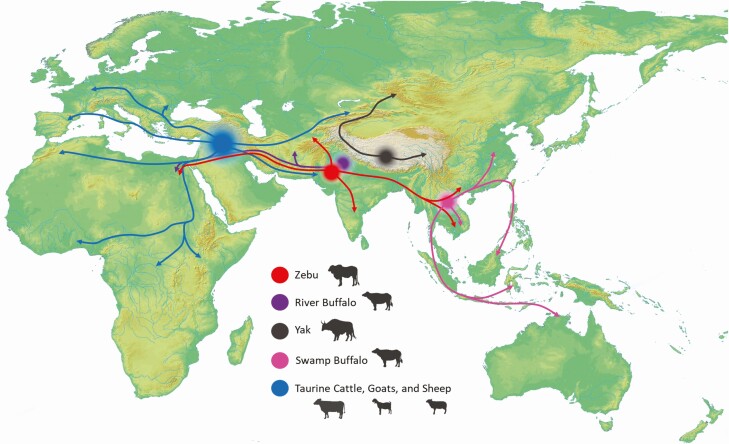
Core regions of ruminant domestication during the early- and mid-Holocene with inferred routes of subsequent dispersal associated with the adoption of farming.

The earliest evidence for the transition to agriculture occurs in the Western regions of the Fertile Crescent during the late Epipaleolithic (ca. 14 500–11 600 calBP, or calibrated radiocarbon years before present) when there is early evidence of the exploitation and processing of wild grains, stone architecture, and a variety of organized site structures. However, the characteristic features of the Neolithic appear in the archaeological record of this region over a long time span from about 20 to 9 thousand years ago (kya), suggesting that the process was gradual (Maher et al., 2012). The domestication of ruminants was an integrated part of the transition to agriculture and is broadly associated with the domestication of local plants and the adoption of foreign domesticates throughout Eurasia and Africa. Figure 1 provides approximate regions of domestication for key milk-producing domestic species and illustrates inferred routes of dispersal from core domestication zones to other regions. The earliest evidence for the active management and subsequent domestication of taurine cattle (*Bos taurus*), goats (*Capra hircus*), and sheep, the predominant species involved in contemporary human dairy consumption, appear by ca. 10 kya in the fertile crescent (parts of modern Iraq, Turkey, Syria, Lebanon, Jordan, Palestine, and Israel), but the domestication of these species was also a gradual process (Zeder, 2008). The domestication of plants and animals occurred independently in different regions of the world throughout the first half of the Holocene, while farming and herding also spread through migration and cultural diffusion (Belwood, 2005). Within Europe, the process was relatively rapid in the south, occurring between 8 to 7.5 kya, where climatic conditions were favorable for Southwest Asian domesticates; however, the process was more gradual in central and northern Europe due to the challenges of establishing crops in regions with a colder climate and reduced growing seasons (Betti et al., 2020). The presence of domestic cattle within the European archeological record appears broadly concurrent with this chronology, being earlier in Southern Europe but with herding slower to establish in central and northern Europe (Ajmone-Marsan et al., 2010). Numerous wild goat species are found throughout Eurasia; however, *Capra hircus* appears to have been domesticated from wild bezoar populations in the fertile crescent in the very early Holocene (Zheng et al., 2020). The domestication of sheep also occurred within the same timescale and region, and there is evidence of convergent genetic characteristics associated with immunity and productivity traits among domestic *Capra* and *Ovis* species (Alberto et al., 2018). While the origins of goat and sheep herding clearly stems from Southwest Asia, the subsequent movement of domestic goats with early farmers, and hybridization with more widely distributed species of wild goats remain poorly resolved.

Agriculture and herding of cattle and goats spread to the Nile Valley and Northern Africa as a relatively complete package from the Levant at ca. 7,500 to 8,000 BP (Belwood, 2005), but there is evidence for independent domestication of domestic zebu cattle (*Bos indicus*) in the Indus Valley on a similar timescale (Ajmone-Marsan et al., 2010). Within South Asia, the earliest evidence for domestic rice is found 9 kya, but full dependence on agricultural subsistence occurs only after 4 kya following the mid-Holocene movement of crops from both Western Eurasia and China (Stevens et al., 2016). The process of transition toward agricultural lifeways differed in other regions. Species of dairy-producing buffalo are found throughout much of Eurasia, and while their origins, domestication, and dispersal are poorly understood, it appears that river buffalo were domesticated in the western Indian Subcontinent and their use subsequently moved westward, while swamp buffalo were domesticated in Southern China on a similar timescale (Zhang et al., 2020). In China, there is evidence for the in situ domestication of millet and rice in the early to mid-Holocene. There appears to be active management of taurine cattle in the early Holocene before the introduction of domestic cattle in the later Holocene (Zhang et al., 2013). Yak were domesticated in the mid-Holocene on the Tibetan Plateau, perhaps as result of hybridization with domestic Taurine cattle (Jacques et al., 2021). Further domestication of plant and animal species occurred in the early and mid-Holocene of the equatorial Africa, the Americas, and Papua New Guinea, but these domestication events are not thought to generally relate to the origins of dairying.

It is important to note, however, that quite separately from the domestication events described earlier, the Neolithic and recent colonial expansions of human populations in the mid-to-late-Holocene, saw the establishment of significant global trade networks and the widespread movement of dairy animals to virtually regions of the planet capable of sustaining herding (Ajmone-Marsan et al., 2010). As a result of the widespread movement, breeding, and herding of mammals, primary and secondary dairy products now form an important component of the diet and cultural diversity of many human populations worldwide. Overall, therefore, the mosaic emergence of different forms of agriculture over recent millennia means that human populations have varied substantially in the degree, timing, and intensity of their association with milk-producing domesticated animals. These cultural transitions led to animal milk and milk-derived products being consumed beyond the age at which consumption of human breastmilk ceases, a dietary shift that likely had not occurred previously within our evolutionary lineage.

## The Evolution of Lactase Persistence in Humans

Humans, like all mammals, produce the enzyme beta-galactosidase (commonly referred to as lactase) during infancy. Lactase serves to cleave dietary lactose into the monosaccharides glucose and galactose which are then absorbed by the small intestine, allowing digestion of the lactose in breastmilk. The concentration of lactose in human breastmilk is relatively high, approximately 7 g/100 mL, and somewhat less in bovine milk, ranging from about 4.6 to 5.0 g/100 mL, but the ability to metabolize lactose can provide a high-caloric content of 2 to 4 kcal/g (Schaafsma, 2008). The domestication of animals was particularly important for early farmers for secondary products such as milk, wool, or labor; however, most humans lose the ability to digest the milk sugar lactose in early childhood leaving them lactose intolerant as adults. Concentrations of lactose in milk can be reduced through fermentation by producing yogurt, or by producing cheese where lactose is removed in whey. Lactose malabsorption is often treated as a medical condition, as it can lead to growth stunting, diarrhea, distention of the abdomen, and decreased appetite (Schaafsma, 2008); however, it is better understood in relation to human adaptation in the past. While nonlactase persistent adults can still consume modest quantities of milk without physiological consequences, the production of cheese or yogurt allows lactose intolerant adults to consume greater quantities of energetically rich dairy products. The presence of milk proteins on pot residues found throughout Europe during the Neolithic attests to the widespread use of milk among early farmers (Evershed et al., 2022), but this evidence does not differentiate whether the milk was directly consumed, or used to produce cheese or yogurt. While there is compelling evidence that dairy products were widely used by Neolithic people, it is likely that in most contexts they used techniques to reduce milk to cheese and yogurt biproducts (Sibbesson, 2019). We can, however, infer spatiotemporal contexts where there was direct reliance on milk consumption from the pattern of evolution in genes associated with lactase persistence (LP).

*LCT* is a protein-coding gene on chromosome 2 that is associated with the production of intestinal lactase. While lactose-intolerant adults may still consume small quantities of raw milk, the failure to cleave lactose molecules into digestible sugars means that lactose remains in the intestine where it has an osmotic effect, of drawing water. Within the large intestine, the lactose molecules fuel bacterial growth which can cause pain and flatulence. A number of single nucleotide polymorphisms have been identified in promotor regions of intron 13 of the *minichromosome maintenance complex component 6* gene, which is upstream of *LCT*. These serve as enhancers of transcriptional activation the *LCT* gene thus promoting the production of intestinal lactase during adulthood. The −13.910:T mutation is found throughout Europe with the greatest frequencies in the North, which is indicative of strong selection in this region over in the last 4,000 years (Evershed et al., 2022). This specific variant is also found in regions of Asia to North Africa, where it likely spreads through gene flow. There is also strong evidence for convergent evolution in LP, through at least four different mutations associated with LP, among specific populations in Eastern and Western Africa, Arabia, and South Asia (Ségurel and Bon, 2017). In all of these regions, selection of LP variants suggests that there were adaptive responses to cultural change involving the direct consumption of milk in the millennia following the adoption of agriculture.

**Figure 2.**
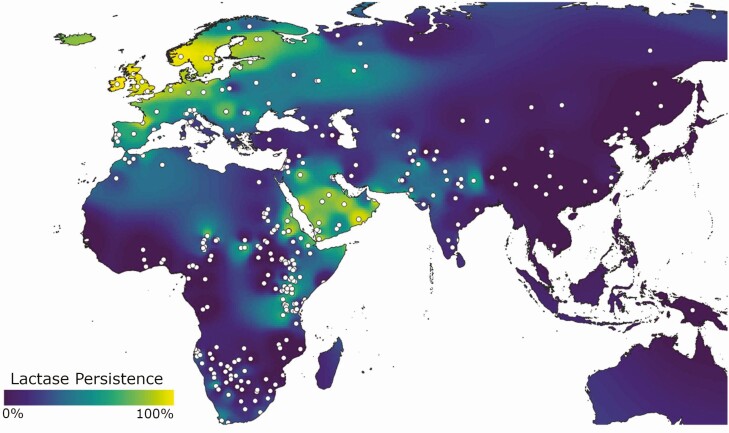
Map of lactase persistence throughout Eurasia and Africa. Adapted from Wells et al. (2021).

There is a general pattern as to where LP phenotypes occur, and this is in either northern latitudes, higher altitudes, or regions subject to periodic droughts, exemplified today in regions such as Northern Europe, Arabia, the Ethiopian and Kenyan Highlands, and parts of the Himalayas (Figure 2). In each of these regions, domestic plant species are either difficult to establish or vulnerable to seasonal variations in climate or precipitation (Stock et al., 2023). The earliest evidence for the direct consumption of milk comes from whey protein recovered from Neolithic (ca. 5,000 BP) dental calculus from Northern Europe (Warinner et al., 2014). In such regions, dairy products likely provided important fallback foods. The large intestine of humans is relatively small, which does not allow for either the space or bacterial communities required for microbial fermentation of many of the fiber components and cellulose found in grasses and plant communities in marginal environments. Herd animals, with their chambered fermenting stomachs, are able to process such sparse and nutritionally deficient plants into high-energy and nutrient-rich secondary products that humans can consume, thus enhancing human dietary adaptation to marginal environments.

Secondary dairy products such as cheese and yogurt are slower to ferment in colder climates, so cultural mechanisms of processing raw milk would be less effective at producing edible dairy products with reduced concentrations of lactose. In combination with the patterns of LP observed, this suggests that direct consumption of raw milk was crucial to human survival in marginal environments. Recent research suggests that the selective pressure related to the evolution of LP in Europe relates to either pathogen exposure or famine (Evershed et al., 2022). Such selection pressures would impose energetic demands on human populations, either through the direct need for nutrition calories to mediate famine, or to fuel the metabolic costs of extended immune function (Wells and Stock, 2020). Our own research suggests that directional selection on LP genes may have been the strongest in regions where agricultural crops were difficult to establish (Stock et al., 2023); however, the biology of milk consumption suggests that its fitness benefits may have gone beyond increasing or stabilizing energy supply.

## Milk Consumption, Nutrition, and Growth

We have previously discussed the high-caloric content of milk, and the role it has in establishing the microbiome, and transferring hormones, proteins, and other nutrients from mothers to offspring. In this context, milk is essentially a superfood that evolved to fuel early life somatic and neural development for infant mammals. What are the consequences of extending the consumption of milk beyond infancy?

Observational studies broadly support the hypothesis that throughout development, consuming animal milk promotes human linear growth. In a US cohort, for example, each additional 236 mL of milk consumed daily was associated with 0.4 cm greater height (Marshall et al., 2018). A number of key questions arise from this observation. Does milk consumption correspond with human growth more broadly through time and space? If so, did this increment in growth have strong enough fitness benefits to have driven selection for LP? Conversely, is variation in human growth a consequence of dependence on milk as a fallback food, and not directly related to selection? Consideration of variation in the consequences of milk consumption on human growth through time and space will help to elucidate these questions. While the milk of all domestic ruminants has relatively similar caloric content, the milk of taurine cows, zebus, buffalos, yaks, goats, and sheep all have somewhat lower lactose content (between 4.4 and 5.1 g/100 g), but generally greater fat, protein, and energy content than human milk (Wijesinha-Bettoni and Bulingame, 2013).

The fitness benefits of the consumption of raw ruminant milk may then have been primarily through greater access to dietary energy which could have been allocated to growth, reproduction, immune function, or other life history functions. One possibility is that drinking milk accelerated the pace of development, via earlier menarche and faster growth in adolescence, thus enabling earlier reproduction (Wiley, 2012). Another possibility is that beyond driving greater stature, the impact of increased hepatic secretion of IGF-1 on skeletal growth, stimulated by the consumption of milk, may have increased the dimensions of the birth canal in adolescent and adult females, thus reducing the risk of obstructed labor, a significant source of maternal mortality even in contemporary human populations (Wells et al., 2021).

Although uncertainties remain, a life history perspective highlights that the capacity to drink milk appears to provide more than just calories, and has a unique impact on both maturation patterns and skeletal dimensions. Opportunities to increase our understanding may derive from testing similar developmental and life history hypotheses across populations among whom LP has been promoted by different mutations.

## The Long-Term Consequences of LP Evolution

When we consider the deep bioarcheological record for herding and dairy consumption among Neolithic peoples throughout Eurasia and Africa, dairying has clearly been a core aspect of human cultural change in many regions throughout the past 10,000 years and has shaped the patterns of variation in the human genome. Are there other long-term consequences of dairying for our species? In three recent articles, we have argued that dairy consumption has a key role in human energetic trade-offs during growth, and as such influences patterns of human growth, phenotypic diversity, and later-life health outcomes (Wells and Stock, 2020). We have argued that the evolution of LP and the associated increase in milk consumption may have “turbo-charged” growth in regions where the transition to agriculture had deleterious effects on human growth and health (Wells et al., 2021). A general decline in human stature associated with the transition to agriculture has generally been widely reported. Our own recent research suggests that a broad reduction in human body size in the terminal Pleistocene and early Holocene actually preceded the adoption of agriculture in many regions (Stock et al., 2023). In that paper, we proposed and evaluated components of the “Lactase Growth Hypothesis” which suggests that LP and the ability to digest milk and lactose increased dietary calories, shifted the energetic biology of human growth, and fuelled regional differences in human body size. Our results noted that in regions of Eurasia characterized by in situ domestication of species and extended periods of mixed foraging and agricultural subsistence, human body size was relatively stable over thousands of years. In contrast, in regions such as Central and Northern Europe where foreign domesticates were difficult to establish and growing seasons were shorter, increases in human body size are correlated with the timing of selective sweeps for LP. These results suggest that the evolution of LP directly influenced geographic patterning of human growth across the past 10,000 years.

**Figure 3.**
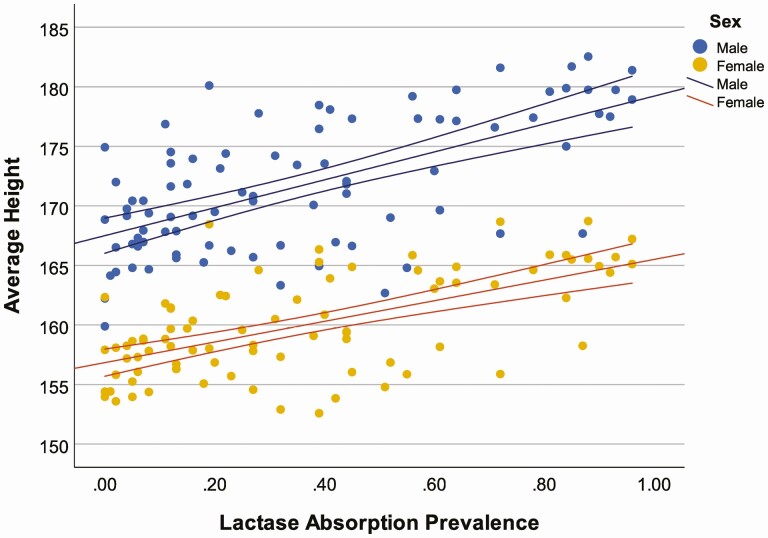
Correlation between average heights and prevalence of lactase absorption in 86 countries (♂: *r*^2^ = 0.389, *P* ≤ 0.001; ♀: *r*^2^ = 0.368, *P* ≤ 0.001).

**Figure 4.**
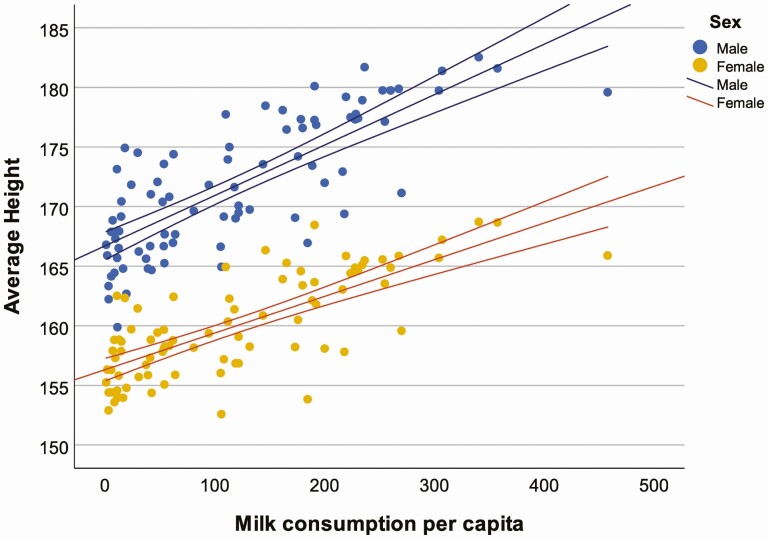
Correlation between average heights and per capita milk consumption (kg/person/yr) in 86 countries (♂: *r*^2^ = 0.611, *P* ≤ 0.001; ♀: *r*^2^ = 0.560, *P* ≤ 0.001).

We further explore the relationship among contemporary populations, by considering the correspondence between global variation in male and female average heights (NCD-RisC, 2017) and: 1) proportions of individuals with lactase absorption as adults (Schaafsma, 2008), and; 2) national level data on milk consumption per capita (United Nations, 2017). The results show a general correlation between mean stature and lactase absorption (Figure 3) for both males (*r*^2^ = 0.389, *P* ≤ 0.001) and females (*r*^2^ = 0.368, *P* ≤ 0.001), and a stronger correspondence with milk consumption per capita (calculated as the food remaining for human use after deduction of all nonfood utilizations, Figure 4) for both males (*r*^2^ = 0.611, *P* ≤ 0.001) and females (*r*^2^ = 0.560, *P* ≤ 0.001). This relationship is likely confounded by other general indicators of health disparity and inequality; however, it is under conditions of nutritional deprivation that we would expect to see a correspondence between body size and LP emerge, if *LCT* genetic variants serve as “energy access” genes. Human height variation, while influenced by many genes, is highly plastic during development (Wells and Stock, 2011). We expect that LP could buffer nutritional stress during development, and when present in conditions of nutritional deprivation and frequent growth stunting, promote growth and lead to differences in body size. Conversely, correspondence between LP phenotypes and body size would not be predicted where there is broad nutritional sufficiency. The correlation between milk consumption and stature reported here should not necessarily be interpreted as a direct causal association as these variables may be correlated to broader societal differences in health care, life-expectancy, or productivity. These results are, however, suggestive of associations between milk consumption and stature that require further investigation.

**Figure 5.**
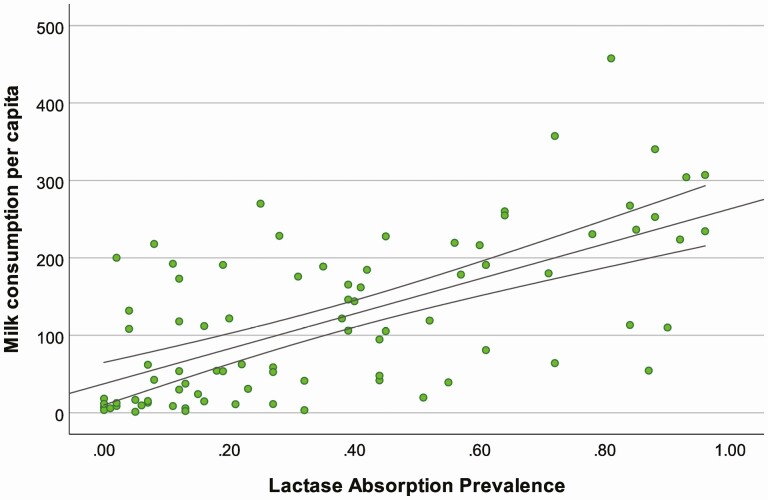
Correlation between per capita milk consumption and lactase absorption prevalence in 86 countries.

We further consider the correspondence between contemporary patterns of milk production per capita and the frequency of LP phenotypes (Figure 5). The results confirm a significant correlation between these variables (*r*^2^ = 0.417, *P* ≤ 0.001). It is not necessarily surprising to identify a relationship between the relative milk production of different economies and the physiological ability of adults in those populations to digest quantities of milk lactose. Further investigation of the relationships between these factors, in relation to nutritional components of national economies, is required.

## Conclusion

In this article, we have briefly reviewed current understandings of the transition to agriculture in Eurasia and Africa with emphasis on the origin and widespread use of herd animals and dairying. There is strong evidence for the convergent evolution of genes that promote LP in different regions of the world including central and northern Europe, eastern and western Africa, and regions of Arabia and South Asia. The pattern of convergent evolution is suggestive that milk played an important role culturally and nutritionally in regions where agricultural crops were difficult to establish or susceptible to environmental fluctuations. The evolution of LP enabled the digestion of milk lactose through the lifespan allowing raw milk to provide an important energetic buffer in marginal environments. This pattern of gene-culture coevolution led to a variety of consequences for human populations, including increased body sizes in regions where LP genes are found in high frequencies. Those patterns of variation were established in the mid-Holocene, approximately ca. 5,000 BP, and persist among human populations today. These results suggest that ancient dietary change shaped human adaptations and the patterns of diversity we see today. Further research across populations with different alleles for LP may help clarify the specific fitness benefits of the capacity to consume milk.

## About the Author

**Jay Stock** is a professor at the Department of Anthropology, Western University, Canada. His research focuses on the influences of cultural change, human behavior, and the natural environment on human biology, primarily interpreted through archeological human remains. This work includes the behavioral influences on the mechanobiology of the human skeleton, human physiology in response to stress, and long-term influences of the transition to agriculture on human biology and health. He received his PhD in Biological Anthropology from the University of Toronto in 2002 and was a lecturer and reader at the University of Cambridge from 2002 until 2019.

**Jonathan Wells** is a professor at UCL Great Ormond Street Institute of Child Health. As a biological anthropologist, his research focuses on birth cohort studies and randomized trials in low- and middle-income countries to elucidate the role of nutrition in the developmental origins of adult health and disease, in order to identify opportunities for nutritional interventions to improve health outcomes. He contributes to the development of evolutionary perspectives, in order to optimize the design of these interventions and interpret the findings. He received his PhD in Biological Anthropology and Human Nutrition from the University of Cambridge and the MRC Dunn Nutrition Unit in 1994 and was lecturer and reader at the UCL Institute of Child Health from 1998 to 2010.
